# Prehospital Trauma Quality Improvement: The Role of Prehospital Database in Improving Timely Access to Trauma Care

**DOI:** 10.1002/wjs.12697

**Published:** 2025-07-08

**Authors:** Lubna Khan, Fayez Aldarsouni, Jalal Alowaisi, Yousef M. Alsofayan, Fahad Alhajaj, Norah Alsubaie

**Affiliations:** ^1^ Center for Global Surgery Baylor College of Medicine Houston Texas USA; ^2^ Department of Trauma Surgery King Saud Medical City Riyadh Saudi Arabia; ^3^ Department of Surgery College of Medicine Imam Mohammad Ibn Saud Islamic University Riyadh Saudi Arabia; ^4^ Department of Emergency Medicine King Saud University Riyadh Saudi Arabia; ^5^ Department of Emergency Medicine College of Medicine and Medical Sciences Qassim University Unaizah Saudi Arabia; ^6^ Department of Surgery King Saud University Medical City King Saud University Riyadh Saudi Arabia

**Keywords:** delay in care, prehospital infrastructure, Riyadh, Saudi Arabia, trauma, trauma centers, undertriage

## Abstract

**Background:**

Prehospital travel time is critical in trauma patients, with delays and undertriage linked to worse outcomes. We hypothesize that implementing a regional prehospital database will reveal trauma distribution patterns and the underlying causes of delays and undertriage.

**Methods:**

We analyzed 102,895 incident reports from 50 prehospital dispatch centers in Riyadh, using data from the Saudi Red Crescent Authority between January 2021 and March 2023. Distribution of dispatch centers as well as trauma and nontrauma centers (NTC) was mapped using QGIS. Network service zone analysis was applied to delineate service areas for trauma centers (TC), based on road networks and speed limits. Dispatch centers were subsequently grouped according to these service zones to evaluate delays (travel times > 60 min to a level 1 or 2 TC) and undertriage to NTC.

**Results:**

The entire city of Riyadh has access to level 1 and 2 TC within 60 min. The East (41.5%) and North (24.9%) regions have the highest trauma burden, with blunt trauma being the most common injury type (68.3%), followed by penetrating trauma (21.1%). Among red criteria cases, 66.8% were transferred to level 1 or 2 TC, with 21.1% experiencing delays (ranging from 9.7% to 57.7% across dispatch centers) and 28.7% (ranging from 7.0% to 64.8% across dispatch centers) undertriaged to nontrauma centers. The highest delays were observed in the 60 min service area (North).

**Conclusion:**

Despite access to TC, delays and undertriage of trauma patients are common, especially in areas with longer travel times. Implementation of prehospital databases can help identify areas with high burden of trauma that are prone to delays and undertriage, thereby improving the timeliness and accuracy of trauma triage.

## Graphical abstract

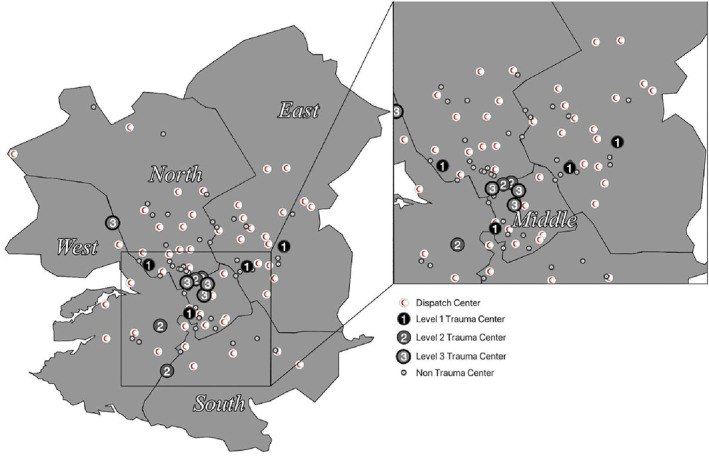

**Abbreviations**
FTTG: Field Trauma Triage Guidelines
NTC: nontrauma center
QGIS: quantum GIS
RC: red criteria
TC: trauma center

## 1 Introduction

Prehospital triage and timely access to trauma care at dedicated trauma centers are crucial for improving outcomes in severely injured patients [1]. Early identification and categorization of critically injured patients through prehospital triage can reduce mortality and improve outcomes [2]. Access to level 1 and level 2 trauma centers within the golden hour is vital, as delays in care, especially for patients requiring immediate intervention, can lead to higher mortality [3]. Despite established trauma protocols and triage systems, transport delays to trauma centers remain common [4]. These delays may go unaddressed because prehospital data are not integrated with trauma registries, which are typically used for quality improvement [5].

National prehospital databases can identify trauma burden and guide quality improvement, especially in countries, such as Saudi Arabia, where road crashes caused 24,000 injuries and 4423 deaths in 2023 alone [6, 7]. The economic impact of these accidents exceeds 14.6 billion US dollars, underscoring the urgency of addressing system‐level barriers to effective trauma care delivery [8, 9, 10]. However, a significant gap remains in the availability of regional prehospital data and in the coordination between prehospital services and trauma centers [11].

This study aims to characterize the overall burden of trauma in Riyadh city by injury mechanism, severity, and geographic location. It also sought to quantify the frequency and patterns of delays and undertriage among high acuity cases to identify opportunities to improve prehospital triage and overall trauma care delivery.

## 2 Methods

### 2.1 Context

Saudi Arabia's national prehospital service, the Saudi Red Crescent Authority (SRCA), is an independent government agency that provides free coverage through the nationwide emergency number 997 [12]. A central command center dispatches 1379 ambulances from 486 stations, 108 of which are in Riyadh [12]. Routine calls are handled by two‐paramedic basic life‐support crews, whereas mobile intensive‐care units add a physician to deliver advanced life support [13, 14]. Since 2020, SRCA has used an electronic decision‐support platform that grades injury severity, recommends the nearest appropriate trauma center, and logs each activation for quality review [12]. Operating under a scoop‐and‐run doctrine, with a median national response time of 10–13 min [12]. National trauma‐destination criteria are being introduced, but most provinces still transport patients to the closest hospital even when definitive trauma care is unavailable [15]. Helicopter transport remains rare because of weather and regulatory constraints but is expected to expand under Vision 2030 healthcare reforms [16].

### 2.2 Data Acquisition, Inclusion and Exclusion Criteria

Data were obtained from the official data registry of the SRCA, as no formal trauma registry exists in the country. A total of 223,540 incident reports from its inauguration in January 2021 to March 2023 were reviewed. The inclusion criteria were as follows: (1) reports originating from Riyadh city and (2) trauma‐related incidents involving patients aged 14 years or older. Reports were excluded if they had missing outcome data (*n* = 73,891), originated from regions outside Riyadh (*n* = 39,574), involved pediatric cases (*n* = 5321 and age < 14), or were nontrauma related (*n* = 1421).

### 2.3 Case Classification and Regional Analysis

Based on official municipal designations, Riyadh city is divided into five distinct regions: North, South, East, West, and Middle. Trauma cases were grouped into four major categories: blunt, penetrating, thermal, and other. Blunt trauma included motor vehicle accidents and falls; penetrating trauma encompassed stab wounds and gunshot injuries; thermal trauma covered flame burns, frostbite, scalds, and other heat or cold exposures; and “other” trauma comprised incidents related to domestic violence, sexual offenses, and other miscellaneous causes. Travel time, defined as the interval from SRCA assignment to hospital arrival, was calculated using dispatch center locations as proxies for incident sites.

### 2.4 Field Trauma Triage Guidelines (FTTG)

Red criteria (RC) cases were defined as follows: all cases of penetrating trauma, for individuals aged 14–64 years, systolic blood pressure (SBP) < 90 mmHg, or heart rate (HR) > SBP); for those aged 65 years and above, SBP < 110 mmHg or HR > SBP.

Any travel time to a level 1 or 2 trauma center (TC) exceeding 60 min was classified as a delay. Undertriage was defined as transfer of RC patients to a level 3 or nontrauma center (NTC) when a level 1 or 2 TC was reachable within 60 min.

### 2.5 Mapping and Statistical Analysis

For visualization, we used Quantum GIS (QGIS) to map trauma center locations based on coordinates from the Riyadh Municipality's official map. We applied network service zone analysis in QGIS to delineate service areas for level 1 and 2 trauma centers reachable within 15, 30, 45, and 60 min. Dispatch centers were categorized by these service areas using precise driving time estimates derived from actual road networks and speed limits to accurately determine theoretical hospital arrival times.

The study was granted Institutional Review Board exemption by King Saud University because only deidentified data were used, and patient consent was not required as the study was retrospective in nature.

## 3 Results

### 3.1 Trauma Burden and Red Criteria Cases

A total of 102,895 trauma cases met the inclusion criteria. Among these, the highest burden was reported in the East (41.5%, *n* = 42,665) and North (24.9%, *n* = 25,576) of Riyadh, followed by the West (12.7%, *n* = 13,053), South (12.1%, *n* = 12,475), and Middle (8.9%, *n* = 9126) (Table 1). Males represented 73.8% (*n* = 75,973) of the cases, with 87.9% (*n* = 90,478) aged 14–64 years. Blunt trauma was the predominant injury mechanism, observed in 68.3% (*n* = 70,304) of cases, primarily driven by motor vehicle collisions (47.9%, *n* = 49,294) and falls (20.4%, *n* = 21,010). Penetrating trauma, thermal injuries, and other trauma accounted for 21.1% (*n* = 21,666), 1.9% (*n* = 1949), and 8.7% (*n* = 8976) of cases, respectively. Hospital transfer data showed that 62.5% (*n* = 64,300) of all trauma cases were managed at a level 1 or 2 trauma center.

**TABLE 1:** Demographics and regional burden of trauma cases.

Parameter	All cases	Red criteria cases
*N* (%)	102,895 (100)	31,072 (30)
Sex		
Male	75,973 (73.8)	20,601 (66.3)
Female	26,109 (25.4)	10,286 (33.1)
*Missing*	813 (0.8)	185 (0.6)
Age in years		
14–64	90,478 (87.9)	22,075 (71.0)
≥ 65 Years old	11,140 (10.8)	8749 (28.2)
*Missing*	1277 (1.2)	248 (0.8)
Type of trauma		
Blunt trauma	70,304 (68.3)	3482 (11.2)
Motor vehicle collisions (MVCs)	49,294 (70.1)^a^	1979 (56.8)^a^
Falls	21,010 (29.9)^a^	1503 (43.2)^a^
Penetrating trauma	21,666 (21.1)	21,666 (69.7)
Thermal trauma	1949 (1.9)	112 (0.4)
Other injuries	8976 (8.7)	165 (0.5)
Location		
East	42,665 (41.5)	13,267 (42.7)
North	25,576 (24.9)	6680 (21.5)
West	13,053 (12.7)	4205 (13.5)
South	12,475 (12.1)	4133 (13.3)
Middle	9126 (8.9)	2787 (9.0)
Arrival hospital designation		
Level 1 and level 2 trauma centers	64,300 (62.5)	20,747 (66.8)
Level 3	5451 (5.3)	1413 (4.5)
Nontrauma center	33,144 (32.2)	8912 (28.7)

^a^ Percentages for MVCs and falls are based on total blunt trauma cases, whereas all other percentages are calculated relative to the total number of all cases or red criteria cases.

Among the 31,072 RC cases, males comprised 66.3% (*n* = 20,601) and 71.0% (*n* = 22,075) were aged 14–64 years. Penetrating trauma was the most common mechanism of injury (69.7%, *n* = 21,666), whereas blunt trauma accounted for 11.2% (*n* = 3482) of RC cases. Regionally, East Riyadh had the highest proportion of RC cases (42.7%, *n* = 13,267), followed by the North (21.5%, *n* = 6680) and West (13.5%, *n* = 4205). Of all RC cases, 66.8% (*n* = 20,747) presented to a level 1 or 2 TC, with 28.7% (*n* = 8912) diverted to NTCs. A detailed description of trauma cases by severity, injury pattern, and regional trauma burden is provided in Supporting Information S1: 1–3.

### 3.2 Distribution of Dispatch Centers and Trauma Care Access

**FIGURE 1:**
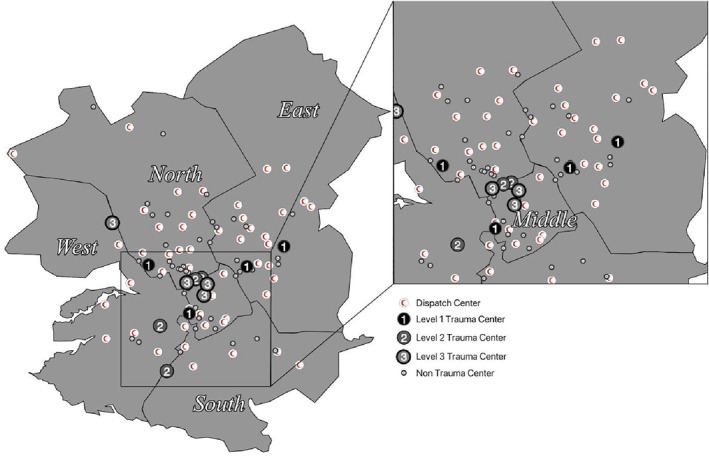
Distribution of dispatch centers, trauma centers (level 1–3), and nontrauma centers in Riyadh, Saudi Arabia.

There are 50 dispatch centers distributed across Riyadh, with 40% (*n* = 20) located in the East and 26% (*n* = 13) in the North, followed by the West (14%, *n* = 7), South (12%, *n* = 6), and Middle (8%, *n* = 4) (Figure 1). Among the 53 hospitals in the city, 15.1% (*n* = 8) are level 1 or 2 trauma centers, 7.5% (*n* = 4) are level 3 centers, and 77.4% (*n* = 41) are NTCs.

**FIGURE 2:**
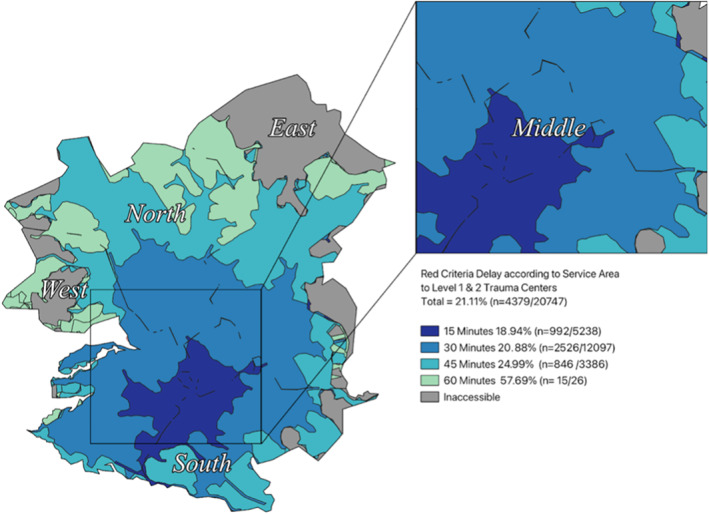
Service area coverage to level 1 or 2 trauma centers at 15, 30, 45, and 60 min and delays of red criteria cases in each service area.

Level 1 and 2 TCs in the West, South, and Middle regions are typically accessible within 15–30 min (Table 2). In the East, access ranges from 15 to 45 min, and in the North, it extends from 30 to 60 min. Despite citywide access to a level 1 or 2 TC within 60 min, 21.1% (*n* = 4379) of RC patients experienced delays (> 60 min). Delays ranged from 18.9% in the 15 min service area to 57.7% in the 60 min service area (Figure 2).

**TABLE 2:** Red criteria (*n* = 31,072) to level 1 and 2 trauma centers.

Region	Accessibility	Total *n* (%)	Delayed *n* (%)
Overall	Total	20,747 (66.8)	4379 (21.1)
East	**Total**	**9278 (44.7)**	**1824 (41.7)**
	15 min	316 (3.4)	78 (4.3)
	30 min	6247 (67.3)	1149 (63.0)
	45 min	2715 (29.3)	597 (32.7)
North	**Total**	**3731 (18.0)**	**938 (21.4)**
	30 min	3034 (81.3)	674 (71.9)
	45 min	671 (18.0)	249 (26.5)
	60 min	26 (0.7)	15 (1.6)
West	**Total**	**3495 (16.8)**	**712 (16.3)**
	15 min	1663 (47.6)	293 (41.2)
	30 min	1832 (52.4)	419 (58.8)
South	**Total**	**2688 (13.0)**	**563 (12.9)**
	15 min	2156 (80.2)	379 (67.3)
	30 min	532 (19.8)	184 (32.7)
Middle	**Total**	**1555 (7.5)**	**342 (7.8)**
	15 min	1103 (70.9)	242 (70.8)
	30 min	452 (29.1)	100 (29.2)

*Note:* Bolded values are total counts for each region.

### 3.3 Red Criteria Delays in Transfers to Level 1–2 Trauma Centers by Dispatch Center

**FIGURE 3:**
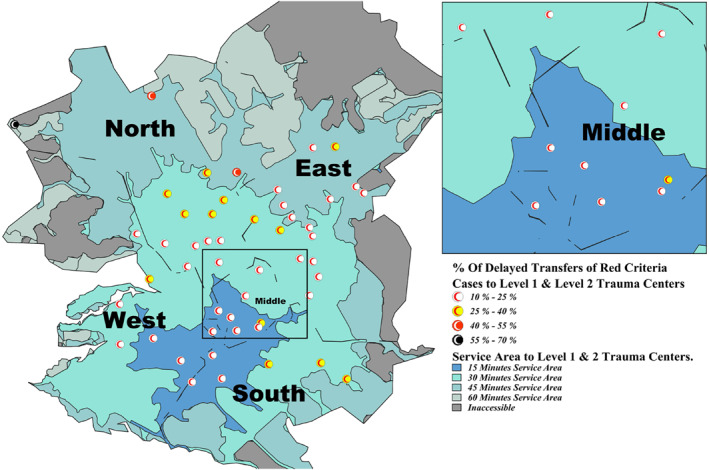
Regional delays (> 60 min) in transfer of red criteria cases to the nearest level 1 and 2 trauma centers with underlay of service area (expected time to nearest trauma center).

Delayed transfers to level 1–2 TCs ranged from 9.7% to 57.7% across all service areas (Figure 3), totaling 21.1% (*n* = 4379/20,747) across the city. In the 15 min area, delays ranged from 9.7% to 29.4%, with Al‐Khalidiya (Middle) having the highest at 29.4% (Table 3). For the 30 min area, delays ranged from 9.7% to 37.6%, with Al‐Kharj (East) showing the highest at 36.4%. In the 45 min area, delays ranged from 20% to 42.4%, with Al‐Amanah (North) and Prince Norah University campus (North) showing delays up to 42.4%. In the 60 min area, Al‐Uyaynah (North) had the highest delay at 57.7%.

**TABLE 3:** Delayed transfer of red criteria (RC) cases to a level 1 and 2 trauma center (TC) and RC transferred to a nontrauma center (NTC) by service area.

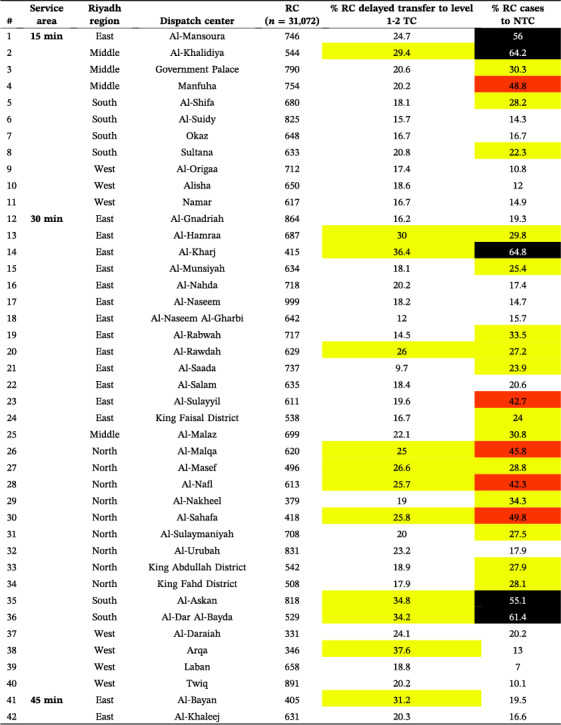
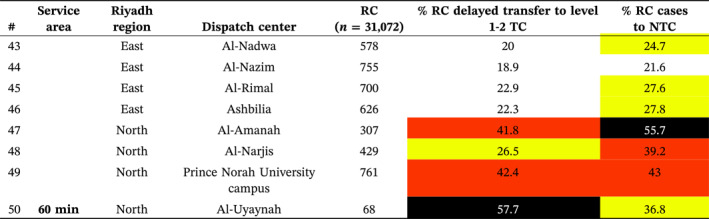

*Note:* Color values correspond to the colors in the map for the various ranges of delays and transfer to non‐trauma centers.

### 3.4 Transfer of Red Criteria Cases to Nontrauma Centers by Dispatch Center

**FIGURE 4:**
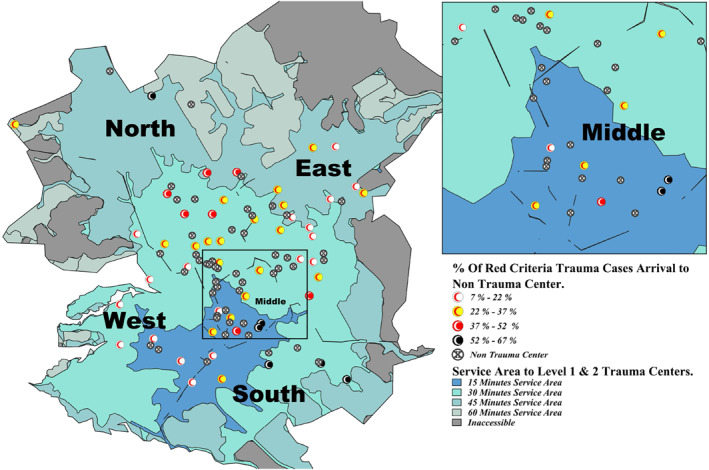
Transfers of red criteria cases by dispatch center to nontrauma centers with underlay of service area (expected time to nearest level 1 or 2 trauma center).

The percentage of red criteria (RC) cases transferred to NTC ranged from 7.0% to 64.8% across all service areas (Figure 4), totaling 28.7% (*n* = 8912/31,072) across the city. In the 15 min area, transfers ranged from 9.7% to 29.4%, with Al‐Khalidiya (Middle) having the highest at 64.2% (Table 3). In the 30 min area, transfers ranged from 7.0% to 49.8%, with Al‐Sahafa (North) showing the highest at 49.8%. In the 45 min area, transfers ranged from 16% to 55.7%, with Al‐Amanah (North) having the highest at 55.7%. In the 60 min area, transfers ranged from 12.2% to 36.8%, with Al‐Uyaynah (North) showing the highest at 36.8%.

## 4 Discussion

Our study demonstrates how a prehospital database can identify system‐level challenges in timely care access, providing data to drive targeted improvements and inform intervention strategies. We found that trauma incidence is unevenly distributed, with the East and North regions bearing a disproportionate burden, primarily of blunt trauma. Although less common, penetrating injuries are more severe, accounting for 69.7% of red criteria cases. Additionally, our spatial analysis revealed significant prehospital delays and undertriage, with up to 57.7% delays in 60 min service areas, and 28.7% of red criteria cases diverted to NTCs.

In Riyadh, the disproportionate injury burden in the East and North, these regions is likely due to heavy traffic, hazardous road conditions, and inadequate speed regulation [17]. Population is densely concentrated within the innermost ring roads, creating chronic congestion that slows ambulance egress [18]. By contrast, the sparsely populated northern and eastern outskirts lie farther from definitive care [19] and are crossed by escarpments, wadis, and incomplete highway links that prolong travel times [20]. These areas are also comparatively less urbanized, consistent with earlier studies linking lower urbanization to elevated collision risk [17, 21, 22]. Seasonal sandstorms and summer temperatures above 45°C can ground helicopters and reduce road visibility, reinforcing the need for redundant transport options [23]. Mitigating these challenges calls for stricter speed‐limit enforcement, targeted expansion of trauma‐center capacity in high‐burden areas, and more efficient EMS triage and dispatch protocols [24, 25, 26, 27]. Incorporating population‐density maps, terrain constraints, and weather patterns into station‐siting, helipad placement, and trauma‐center accreditation would ensure upcoming reforms address both volume‐ and geography‐driven access gaps [28].

Although blunt trauma is more common overall, nearly 70% of red criteria activations involve penetrating injuries. This striking contrast underscores the need for enhanced prehospital triage protocols based on injury severity and mechanism, ensuring those requiring urgent intervention are prioritized [29]. Such streamlined triage can improve resource allocation and minimize treatment delays in this vulnerable population.

Our analysis integrates spatial mapping of trauma centers and dispatch locations, providing a nuanced understanding of how geographic and infrastructural factors impact patient transfers and access to care. Although trauma centers are reachable within 60 min across the city, significant regional transport delays in Riyadh highlight shortcomings in prehospital dispatch and transportation systems. In regions targeted for a 15 min response, delays can approach 30%, whereas in 60 min service areas, they may surpass 57%. The disparities in care access across regions, as seen in Riyadh, emphasize the need for tailored solutions, including optimizing traffic flow, reducing on‐scene time, and streamlining dispatch processes, particularly at centers experiencing the longest delays [30, 31]. Implementing air medical services at strategic locations may also help to overcome geographic barriers and expedite transport [32]. Furthermore, the absence of level 1 or 2 trauma centers in northeast Riyadh presents an opportunity to upgrade select privately owned NTCs or establish a new trauma center in the region.

Another notable finding is that nearly 29% of red criteria cases are diverted to NTCs. Although an undertriage rate of 5% is acceptable [33], our study found rates ranging from 7.0% to 64.8% across dispatch centers, with the highest rates sporadically observed in the 15 , 30 , and 45 min service areas. This variability suggests that factors beyond distance, such as inefficiencies in triage, dispatch protocols, and resource allocation, are contributing to undertriage. Notably, the “stay and play” instead of “scoop and run” approach and the tendency to transport patients to the nearest facility rather than the one offering the highest level of care further exacerbate the issue [34, 35, 36]. Strengthening triage guidelines at dispatch centers with high under‐triage rates, enhancing EMS training, and improving inter‐facility communication could help mitigate these delays and ensure critically injured patients receive appropriate care [37, 38]. Further studies are needed to assess the impact of NTC transfers on mortality and functional outcomes.

Several limitations should be acknowledged. First, our analysis relied exclusively on SRCA incident reports, excluding other modes of patient arrival, which may have led to an underestimation of the true trauma burden. A proportion of patients, particularly those with minor injuries, transported by private means, or with delays in seeking care, may bypass formal EMS activation. Due to the absence of integrated national trauma registries, the extent of this underrepresentation remains unclear, potentially limiting the generalizability of the findings. Second, time‐stamp elements (such as call activation, scene arrival, transport duration, and hospital hand‐over) were recorded inconsistently and could not be analyzed, preventing us from distinguishing transport delays from on‐scene delays. Third, the retrospective design limits causal inference and temporal assessments. Finally, the lack of patient outcomes data precluded evaluation of the impact of transfer delays and undertriage on clinical outcomes.

## 5 Conclusion

Prehospital databases can reveal system level challenges, such as regional variations in trauma incidence, delays in care and undertriage, and facilitate improvements in EMS protocols and prehospital resource allocation. By driving data informed interventions, this approach can help reduce delays and improve trauma care quality in other settings. Future studies should assess the impact of integrating prehospital and hospital databases to examine patient outcomes related to both prehospital and hospital factors.

## Author Contributions

**Lubna Khan:** conceptualization, investigation, writing – original draft, methodology, writing – review and editing. **Fayez Aldarsouni:** investigation, methodology, visualization, software, formal analysis, writing – review and editing, data curation. **Jalal Alowaisi:** supervision, resources, writing – review and editing. **Yousef M. Alsofayan**: writing – review and editing, supervision, resources. **Fahad Alhajaj:** resources, writing – review and editing, supervision. **Norah Alsubaie:** supervision, resources, writing – review and editing, investigation, conceptualization, methodology, project administration.

## Conflicts of Interest

The authors declare no conflicts of interest.

## Supporting information

Supporting Information S1

## Data Availability

The data that support the findings of this study are available from the corresponding author upon reasonable request.

## References

[1] P. A. Cameron , B. J. Gabbe , K. Smith , and B. Mitra , “Triaging the Right Patient to the Right Place in the Shortest Time,” British Journal of Anaesthesia 113, no. 2 (2014): 226–233, 10.1093/bja/aeu231. 24961786

[2] R. D. Lokerman , E. A. J. van Rein , J. F. Waalwijk , et al., “Accuracy of Prehospital Triage of Adult Patients With Traumatic Injuries Following Implementation of a Trauma Triage Intervention,” JAMA Network Open 6, no. 4 (2023): e236805, 10.1001/jamanetworkopen.2023.6805. 37014639 PMC10074221

[3] N. W. Medrano , C. L. Villarreal , M. A. Price , P. J. Bixby , E. M. Bulger , and B. J. Eastridge , “Access to Trauma Center Care: A Statewide System‐Based Approach J Trauma,” Acute Care Surgery 95, no. 2 (2023): 242–248, 10.1097/ta.0000000000004002. 37158782

[4] L. Khan , F. Aldarsouni , J. Alowaisi , et al., “Investigating the Burden of Traumatic Injuries and Access to Trauma Centers in Rural Riyadh,” Journal of Surgical Research 304 (2024): 252–258, 10.1016/j.jss.2024.10.037. 39571463

[5] C. D. Newgard , G. K. Sears , T. D. Rea , et al., “The Resuscitation Outcomes Consortium Epistry‐Trauma: Design, Development, and Implementation of a North American Epidemiologic Prehospital Trauma Registry,” Resuscitation 78, no. 2 (2008): 170–178, 10.1016/j.resuscitation.2008.01.029. 18482792 PMC2562032

[6] Gulf News , “Over 105,000 Traffic Accident Reports Filed in Saudi Arabia in 2023 Gulf News,” 2025, https://gulfnews.com/world/gulf/saudi/over‐105000‐traffic‐accident‐reports‐filed‐in‐saudi‐arabia‐in‐2023‐1.1726240961456?utm.

[7] N. McDonald , N. Little , D. Kriellaars , M. B. Doupe , G. Giesbrecht , and R. T. Pryce , “Database Quality Assessment in Research in Paramedicine: A Scoping Review,” Scandinavian Journal of Trauma, Resuscitation and Emergency Medicine 31, no. 1 (2023): 78, 10.1186/s13049-023-01145-2. 37951904 PMC10638787

[8] H. A. Mohamed , “Estimation of Socio‐Economic Cost of Road Accidents in Saudi Arabia: Willingness‐To‐Pay Approach,” Advances in Management and Applied Economics 5, no. 3 (2015): 1–5.

[9] R. J. Alharbi , V. Lewis , I. Mosley , and C. Miller , “Accident Analysis & Prevention,” Anal Prev 144 (2020): 105653, 10.1016/j.aap.2020.105653. 32629227

[10] A. M. Alferdaus and A. Shaher , “Current Trauma Care System in Saudi Arabia: Literature Review and a Proposed Action Plan Saudi J Health Syst,” Res: Anthropology and Aesthetics 1, no. 4 (2021): 123–133, 10.1159/000519607.

[11] J. Cimino and C. Braun , “Clinical Research in Prehospital Care: Current and Future Challenges Clin,” In Practice 13, no. 5 (2023): 1266–1285, 10.3390/clinpract13050114. PMC10605888 37887090

[12] A. M. Al‐Wathinani , S. M. Alghadeer , Y. S. AlRuthia , et al., “The Characteristics and Distribution of Emergency Medical Services in Saudi Arabia,” Annals of Saudi Medicine 43, no. 2 (2023): 63–69, 10.5144/0256-4947.2023.63. 37031375 PMC10082946

[13] T. AlShammari , P. Jennings , and B. Williams , “Evolution of Emergency Medical Services in Saudi Arabia,” Journal of Emergency Medicine, Trauma and Acute Care 2017, no. 1 (2017): 4, 10.5339/jemtac.2017.4.

[14] Saudi Commission for Health Specialties , Paramedic Specialist Blueprint (SCFHS, 2022), 1–35.

[15] A. Alshamrani , T. Alshammari , S. Sawyer , and B. Williams , “Current State of Trauma Services in Saudi Arabia,” Journal of Emergency Medicine, Trauma and Acute Care 2020, no. 1 (2020): 6, 10.5339/jemtac.2020.6.

[16] Y. Alsofayan , F. Alhajjaj , J. Alowais , N. Aljerian , and S. Alzahrani , “Air Ambulance Services Program in Saudi Arabia: A National Healthcare Transformation Plan in Vision 2030,” Saudi Journal of Emergency Medicine 5 (2024): 61–63, 10.24911/sjemed/72-1702810381.

[17] G. E. Ryb , P. C. Dischinger , G. McGwin , et al., “Degree of Urbanization and Mortality From Motor Vehicular Crashes Ann,” Annals of Advances in Automotive Medicine 56 (2012): 183–190. 23169128 PMC3503419

[18] L. Yaseen , N. Al‐Hosain , I. Shatnawi , et al., Impact of Urban Traffic on Fuel Consumption Leveraging IoT Data: Case Study of Riyadh City (King Abdullah Petroleum Studies and Research Center, 2024), 1–32, KAPSARC Discussion Paper KS‐2024‐DP72.

[19] General Authority for Statistics , Population Estimates Publication 2024 (GASTAT, 2024).1.

[20] General Authority for Statistics , Road Transport Statistics 2022: Media Bulletin (GASTAT, 2023), 1–2.

[21] C. Cabrera‐Arnau , R. Prieto Curiel , and S. R. Bishop , “Uncovering the Behaviour of Road Accidents in Urban Areas,” Royal Society Open Science 7, no. 4 (2020): 191739, 10.1098/rsos.191739. 32431872 PMC7211831

[22] A. Vorko‐Jović , J. Kern , and Z. Biloglav , “Risk Factors in Urban Road Traffic Accidents,” Journal of Safety Research 37, no. 1 (2006): 93–98, 10.1016/j.jsr.2005.08.009. 16516927

[23] N. Middleton , S. S. Kashani , S. Attarchi , M. Rahnama , and S. T. Mosalman , “Synoptic Causes and Socio‐Economic Consequences of a Severe Dust Storm in the Middle East,” Atmosphere 12, no. 11 (2021): 1435, 10.3390/atmos12111435.

[24] A. B. Nathens , G. J. Jurkovich , P. Cummings , et al., “The Effect of Organized Systems of Trauma Care on Motor Vehicle Crash Mortality,” JAMA 283, no. 15 (2000): 1990–1994, 10.1001/jama.283.15.1990. 10789667

[25] Branas C. C. , MacKenzie E. J. , Williams J. C. , et al., “Access to Trauma Centers in the United States,” JAMA 293, no. 21 (2005):2626–2633, 10.1001/jama.293.21.2626. 15928284

[26] S. Bauernschuster and R. Rekers , “Speed Limit Enforcement and Road Safety,” Journal of Public Economics 210 (2022): 104663, 10.1016/j.jpubeco.2022.104663.

[27] E. Z. Alenezi , A. M. AlQahtani , S. F. Althunayan , et al., “Prevalence and Determinants of Road Traffic Accidents in Saudi Arabia,” A Systematic Review Cureus 15, no. 12 (2023): e51205. 38283470 10.7759/cureus.51205 PMC10818129

[28] A. Alamri , “A Smart Spatial Routing and Accessibility Analysis System for EMS Using Catchment Areas of Voronoi Spatial Model and Time‐Based Dijkstra’s Routing Algorithm,” International Journal of Environmental Research and Public Health 20, no. 3 (2023): 1808, 10.3390/ijerph20031808. 36767175 PMC9914634

[29] P. E. Fischer , M. L. Gestring , S. G. Sagraves , et al., “The National Trauma Triage Protocol: How EMS Perspective Can Inform the Guideline Revision Trauma Surg Acute Care,” Open 7, no. 1 (2022): e000879, 10.1136/tsaco-2021-000879. PMC8768919 35128069

[30] H. Pham , Y. Puckett , and S. Dissanaike , “Faster On‐Scene Times Associated With Decreased Mortality in Helicopter Emergency Medical Services (HEMS) Transported Trauma Patients Trauma,” Trauma Surgery & Acute Care Open 2, no. 1 (2017): e000122, 10.1136/tsaco-2017-000122. 29766113 PMC5887760

[31] Y. Siripakarn , L. Triniti , and W. Srivilaithon , “Association of Scene Time With Mortality in Major Traumatic Injuries Arrived by Emergency Medical Service,” Journal of Emergencies, Trauma, and Shock 16, no. 4 (2023): 156–160. 38292276 10.4103/jets.jets_35_23 PMC10824223

[32] A. D. Mitchell , J. M. Tallon , and B. Sealy , “Air versus Ground Transport of Major Trauma Patients to a Tertiary Trauma Centre: A Province‐Wide Comparison Using TRISS Analysis Can J,” Surgery 50, no. 2 (2007): 129–133. PMC2384270 17550717

[33] American College of Surgeons Committee on Trauma , Resources for Optimal Care of the Injured Patient (American College of Surgeons, 2014).

[34] R. M. Smith and A. K. T. Conn , “Prehospital Care − Scoop and Run or Stay and Play?,” supplement Injury 40, no. 4 (2009): S23‐S26–S26, 10.1016/j.injury.2009.10.033. 19895949

[35] Z. Kmietowicz , “In Cases of Serious Injury “Scoop and Run”,” Improves Survival Compared With Ambulance BMJ 358 (2017): j4430, 10.1136/bmj.j4430.

[36] B. Haas , T. A. Stukel , D. Gomez , et al., “The Mortality Benefit of Direct Trauma Center Transport in a Regional Trauma System: A Population‐Based Analysis J Trauma,” Acute Care Surgery 72, no. 6 (2012): 1510–1515: discussion 1515‐1517. 10.1097/TA.0b013e318252510a 22695414

[37] M. E. Harrigan , P. A. Boremski , B. R. Collier , A. N. Tegge , and J. R. Gillen , “Impact of Nonphysician, Technology‐Guided Alert Level Selection on Rates of Appropriate Trauma Triage in the United States: A Before and After Study,” Journal of Trauma and Injury 36, no. 3 (2023): 231–241, 10.20408/jti.2023.0020. 39381695 PMC11309284

[38] N. M. Szary , A. Sarwal , B. J. Boshard , et al., “Transfer of Care Communication: Improving Communication During Inter‐Facility Patient Transfer,” Mo Med 107, no. 2 (2010): 127–130. 20446521 PMC6188263

