# Supplementary material for: Prehospital Trauma Quality Improvement: The Role of Prehospital Database in Improving Timely Access to Trauma Care
Source: World J Surg. 2025 Jul 8;49(8):2246–54. doi: 10.1002/wjs.12697 (PMC12338412; doi:10.1002/wjs.12697)
Supplement: Supplementary file 1 — Supporting Information S1 [file WJS-49-2246-s001.docx]

| **Supplement 1. Distribution of Trauma Cases by Injury Severity and Mechanism** | | **All Cases** | | **Falls** | | **Motor Vehicle Accidents** | | **Penetrating** | **Thermal Injuries** | | **Other** | |
| --- | --- | --- | --- | --- | --- | --- | --- | --- | --- | --- | --- | --- |
|  |  | **Total**^†^ **(n, %)** | **Red Criteria (RC)**^‡^ **(n, %)** | **Total**^†^ **(n, %)** | **RC**^‡^ **(n, %)** | **Total**^†^ **(n, %)** | **RC**^‡^ **(n, %)** | **RC (n, %)**^†^ | **Total**^†^ **(n, %)** | **RC**^‡^ **(n, %)** | **Total**^†^ **(n, %)** | **RC**^‡^ **(n, %)** |
| **Total (n, %)** | | **102,895 (100)** | **31,072 (30.2)** | **21,010 (20.4)** | **3,795 (18.1)** | **49,294 (47.9)** | **4,987 (10.1)** | **21,666 (21.1)** | **1949 (1.9)** | **285 (14.6)** | **8976 (8.7)** | **339 (3.8)** |
| **Sex** | Male | 75,973 (73.8) | 20,601 (66.3) | 13,447 (64.0) | 2,062 (54.3) | 40,000 (81.1) | 4,262 (85.5) | 13,898 (64.1) | 1222 (62.7) | 165 (57.9) | 7406 (82.5) | 214 (63.1) |
|  | Female | 26,109 (25.4) | 10,286 (33.1) | 7,405 (35.2) | 1,733 (45.7) | 8,931 (18.1) | 722 (14.5) | 7,586 (35.0) | 705 (36.2) | 120 (42.1) | 1482 (16.5) | 125 (36.9) |
|  | *Missing* | 813 (0.8) | 185 (0.6) | 158 (0.8) | 0 (0) | 363 (0.7) | 3 (0.1) | 182 (0.8) | 22 (1.1) | 0 (0.0) | 88 (1.0) | 0 (0.0) |
| **Age** | ≥65 Years old | 11,140 (10.8) | 8,749 (28.2) | 4,234 (20.2) | 2,336 (61.6) | 669 (1.4) | 398 (8.0) | 5,704 (26.3) | 130 (6.7) | 93 (32.6) | 403 (4.5) | 218 (64.3) |
|  | 14–64 | 90,478 (87.9) | 22,075 (71.0) | 16,551 (78.8) | 1,454 (38.3) | 47,987 (97.3) | 4,582 (91.9) | 15,727 (72.6) | 1756 (90.1) | 191 (67.0) | 8457 (94.2) | 121 (35.7) |
|  | *Missing* | 1,277 (1.2) | 248 (0.8) | 225 (1.1) | 5 (0.1) | 638 (1.3) | 7 (0.1) | 235 (1.1) | 63 (3.2) | 1 (0.4) | 116 (1.3) | 0 (0.0) |
| **Location** | East | 42,665 (41.5) | 13,267 (42.7) | 8,455 (40.2) | 1,503 (39.6) | 19,617 (39.8) | 1,979 (39.7) | 9,508 (43.9) | 824 (42.3) | 112 (39.3) | 4261 (47.5) | 165 (48.7) |
|  | Middle | 9,126 (8.9) | 2,787 (9.0) | 1,814 (8.6) | 306 (8.1) | 4,152 (8.4) | 408 (8.2) | 2,009 (9.3) | 236 (12.1) | 39 (13.7) | 915 (10.2) | 25 (7.4) |
|  | North | 25,576 (24.9) | 6,680 (21.5) | 5,874 (28.0) | 994 (26.2) | 13,215 (26.8) | 1,234 (24.7) | 4,309 (19.9) | 423 (21.7) | 63 (22.1) | 1755 (19.6) | 80 (23.6) |
|  | South | 12,475 (12.1) | 4,133 (13.3) | 2,275 (10.8) | 420 (11.1) | 5,952 (12.1) | 622 (12.5) | 3,023 (14.0) | 228 (11.7) | 36 (12.6) | 997 (11.1) | 32 (9.4) |
|  | West | 13,053 (12.7) | 4,205 (13.5) | 2,592 (12.3) | 572 (15.1) | 6,358 (12.9) | 744 (14.9) | 2,817 (13.0) | 238 (12.2) | 35 (12.3) | 1048 (11.7) | 37 (10.9) |
| **Under-triage**  **(RC transfer to level 3 or non-trauma center)** | | - | 10,325 (33.2) | - | 1,600 (42.2) | - | 2,036 (40.8) | 6,486 (29.9) | - | 80 (28.1) | - | 123 (36.3) |

^(†)^ Percentages indicate each injury mechanism’s share of all 102,895 cases.

^(‡)^ Percentages indicate the proportion of cases that met Red Criteria within each mechanism of injury.

***Note:*** *Because all penetrating injuries were classified as Red Criteria, the percentages reported for penetrating trauma represent their share of the entire cohort rather than the within-mechanism percentage (which would always be 100%).*

| **Supplement 2. Case distribution by Region.** | **All Cases** | **East** | **Middle** | **North** | **South** | **West** |
| --- | --- | --- | --- | --- | --- | --- |
| **N (%)** | **102,895 (100)** | 42,665 (41.5) | 9,126 (8.9) | 25,576 (24.9) | 12,475 (12.1) | 13,053 (12.7) |
| **Red Criteria** | **31,072 (30)** | 13,267 (42.7) | 2,787 (9.0) | 6,680 (21.5) | 4,133 (13.3) | 4,205 (13.5) |
| **Sex** |  |  |  |  |  |  |
| Male | 75,973 (73.8) | 31,844 (74.6) | 7,215 (79.1) | 18,061 (70.6) | 9,412 (75.4) | 9,441 (72.4) |
| Female | 26,109 (25.4) | 10,445 (24.5) | 1,890 (20.7) | 7,310 (28.6) | 2,968 (23.8) | 3,496 (26.8) |
| *Missing* | 813 (0.8) | — | — | — | — | — |
| **Age in years** |  |  |  |  |  |  |
| 14–64 | 90,478 (87.9) | 37,415 (87.7) | 8,169 (89.5) | 22,700 (88.8) | 10,860 (87.1) | 11,334 (86.8) |
| ≥65 Years old | 11,140 (10.8) | 4,667 (10.9) | 891 (9.8) | 2,561 (10.0) | 1,469 (11.8) | 1,552 (11.9) |
| *Missing* | 1,277 (1.2) | — | — | — | — | — |
| **Type of Trauma** |  |  |  |  |  |  |
| Blunt Trauma | 70,304 (68.3) | 28,072 (65.8) | 5,966 (65.4) | 19,089 (74.6) | 8,227 (65.9) | 8,950 (68.5) |
| Motor Vehicle Collisions | 49,294 (47.9) | 19,617 (39.8) | 4,152 (8.4) | 13,215 (26.8) | 5,952 (12.1) | 6,358 (12.9) |
| Falls | 21,010 (20.4) | 8,455 (40.2) | 1,814 (8.6) | 5,874 (28.0) | 2,275 (10.8) | 2,592 (12.3) |
| Penetrating Trauma | 21,666 (21.1) | 9,508 (43.9) | 2,009 (9.3) | 4,309 (19.9) | 3,023 (14.0) | 2,817 (13.0) |
| Thermal Trauma | 1,949 (1.9) | 824 (42.3) | 236 (12.1) | 423 (21.7) | 228 (11.7) | 238 (12.2) |
| Other Injuries | 8,976 (8.7) | 4,261 (47.5) | 915 (10.2) | 1,755 (19.6) | 997 (11.1) | 1,048 (11.7) |
| **Arrival Hospital Designation** |  |  |  |  |  |  |
| Level 1 and Level 2 trauma centers | 64,300 (62.5) | 28,140 (65.9) | 4,339 (47.5) | 13,413 (52.4) | 7,605 (60.9) | 10,803 (82.8) |
| Level 1 | 42,573 (41.4) | 23,614 (55.4) | 2,846 (31.2) | 9,796 (38.3) | 2,175 (17.4) | 4,142 (31.7) |
| Level 2 | 21,727 (21.1) | 4,526 (10.6) | 1,493 (16.4) | 3,617 (14.1) | 5,430 (43.5) | 6,661 (51.0) |
| Level 3 | 5,451 (5.3) | 961 (2.3) | 139 (1.5) | 3,354 (13.1) | 197 (1.6) | 800 (6.1) |
| Non-Trauma Center | 33,144 (32.2) | 13,564 (31.8) | 4,648 (50.9) | 8,809 (34.4) | 4,673 (37.5) | 1,450 (11.1) |

| **Supplement 3. Distribution of Trauma Type across Riyadh City Regions.** | | | | |
| --- | --- | --- | --- | --- |
| **Riyadh city region** | **Dispatch Center** | **All trauma (n=102,895)** | **Blunt Trauma (n=70,304)** | **Penetrating Trauma (n=21,666)** |
| East | Al-Mansoura | 2188 | 1344 | 575 |
|  | Al-Saada | 2445 | 1567 | 542 |
|  | Al-Naseem Al-Gharbi | 1576 | 910 | 479 |
|  | Al-Rabwah | 2333 | 1603 | 458 |
|  | Al-Gnadriah | 2789 | 1839 | 632 |
|  | King Faisal District | 1536 | 928 | 427 |
|  | Al-Munsiyah | 1637 | 1068 | 454 |
|  | Al-Naseem | 2616 | 1560 | 736 |
|  | Al-Salam | 1605 | 890 | 487 |
|  | Al-Sulayyil | 2355 | 1587 | 444 |
|  | Al-Nahda | 2318 | 1507 | 528 |
|  | Al-Rawdah | 2475 | 1698 | 430 |
|  | Al-Hamraa | 2031 | 1385 | 465 |
|  | Al-Kharj | 1523 | 1054 | 260 |
|  | Al-Nazim | 2766 | 1897 | 549 |
|  | Al-Nadwa | 1590 | 956 | 416 |
|  | Al-Khaleej | 2222 | 1502 | 446 |
|  | Ashbilia | 2141 | 1430 | 448 |
|  | Al-Rimal | 2594 | 1841 | 490 |
|  | Al-Bayan | 1925 | 1506 | 242 |
| Middle | Manfuha | 2174 | 1385 | 538 |
|  | Government Palace | 2295 | 1449 | 540 |
|  | Al-Khalidiya | 2465 | 1716 | 413 |
|  | Al-Malaz | 2192 | 1416 | 518 |
| North | King Fahd District | 1698 | 1166 | 373 |
|  | King Abdullah District | 1772 | 1219 | 408 |
|  | Al-Nakheel | 1960 | 1565 | 227 |
|  | Al-Sulaymaniyah | 2584 | 1874 | 464 |
|  | Al-Urubah | 2707 | 1942 | 561 |
|  | Al-Malqa | 2210 | 1651 | 391 |
|  | Al-Nafl | 2203 | 1626 | 393 |
|  | Al-Sahafa | 1791 | 1382 | 250 |
|  | Al-Masef | 2007 | 1494 | 350 |
|  | Al-Narjis | 1762 | 1317 | 265 |
|  | Al-Amanah | 1417 | 1133 | 167 |
|  | Prince Norah University campus | 3087 | 2402 | 424 |
|  | Al-Uyaynah | 378 | 318 | 36 |
| South | Al-Suidy | 2490 | 1652 | 607 |
|  | Okaz | 2247 | 1568 | 454 |
|  | Al-Shifa | 2118 | 1383 | 502 |
|  | Sultana | 1766 | 1130 | 490 |
|  | Al-Dar Al-Bayda | 1493 | 948 | 361 |
|  | Al-Askan | 2361 | 1546 | 609 |
| West | Namar | 1682 | 1074 | 447 |
|  | Al-Origaa | 2122 | 1431 | 506 |
|  | Alisha | 1689 | 1017 | 478 |
|  | Laban | 2250 | 1587 | 425 |
|  | Twiq | 2586 | 1744 | 566 |
|  | Al-Daraiah | 1528 | 1231 | 181 |
|  | Arqa | 1196 | 866 | 214 |
